# Efficacy and safety of Jinghua Weikang capsule combined with amoxicillin-furazolidone triple/quadruple therapies in the rescue treatment of *Helicobacter pylori* infection

**DOI:** 10.3389/fmed.2025.1531620

**Published:** 2025-03-25

**Authors:** Yao Yang, Xin Deng, Hui-Xia Xiao, Su-Man Ye, Zi-Cheng Wang, Feng Jiang, Hai-Xiao Han, Zai-Jian Wang, Ji-Zheng Ma, Yu Lan, Hui Ye, Xue-Zhi Zhang

**Affiliations:** ^1^Department of Integrated Traditional Chinese and Western Medicine, Peking University First Hospital, Beijing, China; ^2^Institute of Integrated Traditional Chinese and Western Medicine, Peking University, Beijing, China; ^3^Kunming Traditional Chinese Medicine Hospital, Kunming, China; ^4^Taikang Xianlin Drum Tower Hosipital, Kunming, China; ^5^Beijing University of Chinese Medicine, Beijing, China; ^6^Dongzhimen Hosipital Beijing University of Chinese Medicine, Beijing, China; ^7^Oriental Hospital of Beijing University of Chinese Medicine, Beijing, China; ^8^Beijing University of Chinese Medicine Third Affiliated Hospital, Beijing, China; ^9^Guang'anmen Hospital, China Academy of Traditional Chinese Medicine, Beijing, China; ^10^Beijing Jishuitan Hospital, Beijing, China

**Keywords:** rescue treatment, bismuth-containing quadruple therapy, multi-center randomized controlled study, Jinghua Weikang capsule, *Helicobacter pylori*

## Abstract

**Aim:**

To evaluate the efficacy and safety of Jinghua Weikang Capsule (JWC) combined with amoxicillin-furazolidone triple/quadruple therapies in the rescue treatment of drug-resistant *H. pylori* infection.

**Methods:**

Patients who failed *H. pylori* eradication therapy at least once were enrolled and randomly assigned into four groups (1:1:1:1), as follows: The control group received 20 mg rabeprazole, 1,000 mg amoxicillin, 220 mg bismuth potassium citrate, and 100 mg furazolidone twice daily (b.i.d.) for 14 days; Group A received 240 mg JWC b.i.d. combined with 20 mg rabeprazole, 1,000 mg amoxicillin, and 100 mg furazolidone b.i.d. for 14 days; Group B received the same regimen as Group A for 14 days, followed by an additional 14 days of 240 mg JWC b.i.d.; and Group C received 240 mg JWC b.i.d. combined with 20 mg rabeprazole, 1,000 mg amoxicillin, 220 mg bismuth potassium citrate, and 100 mg furazolidone b.i.d. for 10 days. The primary outcome was *H. pylori* eradication at 4 weeks after treatment.

**Results:**

Four hundred eighty-eight patients were included in this study. The intention-to-treat (ITT) eradication rates in the four groups were 85.2, 73.8, 78.7 and 75.4% (*p* = 0.136), while the modified intention-to-treat (MITT) eradication rates were 92.0, 84.9, 88.9 and 86.8% (*p* = 0.398), respectively. And the per-protocol (PP) eradication rates were 92.5, 85.4, 87.9 and 86.7% (*p* = 0.405), respectively. The eradication rates were comparable among the four groups. No statistically significant differences in eradication rates were observed between each of the three treatment groups and the control group (all *p* > 0.05). The eradication rate of *H. pylori* in group B demonstrated non-inferiority compared with the control group (*p* = 0.0415; 90% CI, −0.0965 to 0.0336). The four groups exhibited similar frequencies of overall adverse events (9.84, 5.74, 6.56%, 2.46%, *p =* 0.112).

**Conclusion:**

The eradication rate of the JWC-containing regimen demonstrated no statistically significant difference compared with bismuth-containing quadruple therapy in the rescue treatment of *H. pylori* infection. The prolonged JWC treatment regimen exhibited non-inferiority in eradication rates. JWC-containing therapies can effectively reduce the incidence of adverse reactions and significantly alleviate certain clinical symptoms.

**Clinical trial:**

https://clinicaltrials.gov/, identifier ChiCTR1800019326.

## Introduction

*Helicobacter pylori* infection has become a global public health issue due to more than half of the natural population in the world is infected with *H. pylori* (1). *H. pylori* infection can lead to peptic ulcers, chronic gastritis, gastric cancer, and some extra–digestive tract diseases, such as iron deficiency anemia and vitamin B_12_ deficiency (2–7). In China, not only is the infection rate of *H. pylori* high (8), but the incidence and mortality of stomach cancer causes it to rank among the top three cancers (9). Eradication of *H. pylori* can effectively reduce the risk of stomach cancer (10, 11). In many countries worldwide, the efficacy of clarithromycin triple therapy to eradicate *H. pylori* infection has been reduced to less than 80% (12), the proportion of multiple treatment failures and refractory infections has increased, and the increase in the antibiotic resistance rate is the main reason for the failure of *H. pylori* eradication (13). Moreover, the situation of *H. pylori* resistance in different countries and regions also varies (14, 15). The Sixth Maastricht Consensus recommends that bismuth or non-bismuth quadruple therapy should be used as a first-line eradication regimen for 14 days in areas with high clarithromycin resistance (>15%) (16). In China, bismuth quadruple therapy is recommended as the first or mainstream rescue treatment for *H. pylori* eradication (17).

Patients who have previously failed radical treatment of *H. pylori* face the dilemma of gradually reducing the types of antibiotics available due to secondary drug resistance (18, 19), and the eradication effect has had difficulty reaching the expected value. In addition, the bismuth included in quadruple therapy accumulates in the body, which can cause adverse reactions affecting liver and kidney function and the nervous system, resulting in uremia and memory loss (20). Therefore, it is necessary to seek a safer and more effective plan for the clinical remedy and treatment of *H. pylori* infection.

In recent years, the role of traditional Chinese medicine in the treatment of *H.pylori*-related diseases has garnered increasing attention. Its components are relatively straightforward and undergo stringent quality control measures. Among clinically applied Chinese patent medicines, it serves as a convenient avenue for identifying effective drugs to combat *H. pylori* infection. The development of JWC was guided by traditional Chinese medicine theory and based on empirical knowledge, specifically designed for the treatment of peptic ulcer (PU) and chronic gastritis (CG). It consists of a specific blend of essential oils extracted from Chenopodiaceae plant Nemethoria and rubidiaceae plant hydrangea. *In vitro* experiments show that JWC has antibacterial and bactericidal effects against *H. pylori* sensitive and resistant strains, and it has a synergistic effect when used in combination with antibiotics (21, 22). Clinical studies have also shown that the combination of JWC with antibiotics can reduce antibiotic resistance, improve the eradication rate of *H. pylori* infections, lower the incidence of adverse reactions, and reduce the rate of *H. pylori* infection recurrence (23–25). Therefore, based on previous studies, patients who failed to eradicate *H. pylori* with previous treatment were included in this study, and JWC was combined with triple therapy to evaluate the effectiveness and safety of different salvage treatment regimens that include JWC, in order to benefit more *H. pylori*-infected patients.

## Materials and methods

### Study design

This was a prospective, multicenter, randomized controlled, non-inferior clinical study with the main objective of evaluating the *H. pylori* eradication rate of rescue therapy containing JWC compared with that of bismuth quadruple therapy.

The study was conducted from January 2019 to December 2022 at Peking University First Hospital, Guang’anmen Hospital of China Academy of Chinese Medical Sciences, Beijing Jishuitan Hospital, Dongzhimen Hospital Beijing University of Chinese Medicine, Dongfang Hospital Beijing University of Chinese Medicine, and Beijing University of Chinese Medicine Third Affiliated Hospital. Patients with *H. pylori* infection that had failed to be eradicated by quadruple therapy and necessitated rescue treatment were enrolled. Demographic and clinical data of all patients were recorded at the time of enrollment.

The experiment used a random block design. After sequential numbering at each center, patients were randomly assigned in a 1:1:1:1 ratio to a positive control group receiving furazolidone with a bismuth quadruple 14-day regimen and to three treatment groups receiving JWC. During follow-up visits, adverse reactions and medications were recorded. All participants were examined via a urea breath test 4 weeks after the end of eradication therapy to assess *H. pylori* eradication rates. The symptoms of patients were assessed at baseline, at the end of the treatment period, and 1 month, 3 months, and 6 months after the end of treatment.

### Participants

The inclusion criteria were as follows: (1) patients who ranged in age from 18 to 70 years old; (2) patients who had previously experienced treatment failure 1–3 times under quadruple therapy; (3) patients who had discontinued treatment for at least 3 months; (4) furazolidone has not been administered in prior therapeutic protocols; (5) patients who had undergone gastroscopy or imaging examinations within the past 2 years to exclude malignant gastric lesions were included.; (6) patients who had a current *H. pylori* infection; (7) patients who agreed to sign the informed consent forms.

The exclusion criteria were as follows: (1) constant use of antibiotics (against infection under any circumstances), bismuth, PPIs or H2-receptor antagonists within 4 weeks before the *H. pylori* assessment; (2) history of peripheral neuropathy; (3) allergies to the drug used in this study; (4) pregnancy and lactation; (5) participation in other clinical trials during the previous 3 months; (6) severe or unstable cardiopulmonary or endocrine diseases or severe substantial organ impairments and complications; (7) inability to express oneself properly to cooperate.

Stop/exit/exclusion criteria: (1) deterioration or severe complications; (2) severe adverse reactions occur during treatment and cannot be tolerated; (3) other diseases interfered with observation during treatment; (4) The patient does not cooperate and changes the medication regimen at will; (5) lost to follow-up; (6) pregnancy during treatment; (7) The patients voluntarily asked to withdraw from the study; (8) Any situation or condition that puts the subjects at significant risk.

### Intervention

Eligible patients at each center were randomly divided into four groups, and the intervention regimens were as follows: (i) control group: rabeprazole (Jumpcan Pharma, Taixing, Jiangsu) 20 mg, amoxicillin (Zhuhai United Laboratories Co) 1,000 mg, bismuth potassium citrate (Livzon Group Livzon Pharmaceutical Factory, Zhuhai, Guangdong) 220 mg and furazolidone (Chifeng Mengxin Pharm, Chifeng, Neimenggu) 100 mg twice a day (b.i.d.) for 14 days; (ii) group A: JWC (Tianjin Tasly Pharm, Tianjing) 240 mg b.i.d. was taken instead of bismuth in the control group for 14 days; (iii) group B: receiving the same dose as group A for the first 14 days plus JWC 240 mg b.i.d. for another 14 days; and (iv) group C: amoxicillin-furazolidone quadruple therapy and JWC 240 mg b.i.d. were used together for 10 days. Amoxicillin and furazolidone were taken after meals, while other drugs were taken before meals.

### Diagnosis of *Helicobacter pylori* infection

Detection of *H. pylori* infection was completed by the ^13^C test (^13^C-UBT), rapid urease test (RUT), *H. pylori* stool antigen test (HpSAT), or histological examination. At least one positive result for the above tests was confirmed as a present *H. pylori* infection, and a borderline result for ^13^C (DOB:4 ± 2‰) needed to be rechecked.

The ^13^C-UBT was rechecked at least 1 month after the last treatment, and a negative result was defined as *H. pylori* eradication. The diagnostic threshold for a negative ^13^C-UBT result was established at DOB < 4‰, consistent with current international guidelines. The borderline result for ^13^C-UBT (DOB:4 ± 2‰) needed to be rechecked. Patients who have successfully eradicated *H. pylori* should undergo a repeat *H. pylori* test 6 months after the completion of eradication therapy.

### Safety, symptom improvement and compliance

Subjects were informed of possible adverse drug reactions before taking the drugs and were asked to record any abnormal sensations on the diary card. Adverse reactions and serious adverse events: Special attention should be paid to furazolidone-related adverse reactions, including dizziness, nausea, rash, fever, polyneuritis, and even psychiatric disorders during medication. All adverse events (AEs) were documented from occurrence to resolution throughout the study period. Serious adverse events (SAEs) were defined as any of the following outcomes attributed to the investigational drug: death, permanent or severe disability, functional impairment, hospitalization, or life-threatening conditions. All SAEs occurring during the trial were reported to the Medical Ethics Committee of Peking University First Hospital and the National Medical Products Administration (NMPA) within 24 h. A standardized SAE report form was completed, and relevant stakeholders (e.g., sponsors, regulatory authorities) were notified immediately. In emergency situations, investigators promptly implemented appropriate management measures. Adverse Reaction Incidence Rate was calculated as: Incidence (%) = (Number of participants with adverse reactions/Total number of participants receiving medication) × 100.

The clinical symptom score was evaluated by the symptom severity index. Symptom information was recorded at baseline, after the end of eradication therapy, and 4 weeks after the end of eradication therapy. The quantitative criteria of symptom grading were evaluated according to the “guiding principles for Clinical Research of Piman syndrome (chronic gastritis and dyspepsia)” in the “Guiding Principles for Clinical Research of New Chinese Medicine” (26). The severity and frequency of clinical symptoms were divided into none, mild, moderate and severe, and the corresponding scores were 0, 1, 2, and 3. Symptom scores were calculated as follows: symptom score = symptom degree score × frequency score. The total symptom score was calculated as the sum of the scores for all symptoms.

Each participant was provided with a paper medication diary card and a caution card to remind them not to miss or take the wrong medication (Supplementary Table 2). During the medication period, the research assistant actively and timely solved the patients’ questions and informed the patients to review to reduce the dropout rate and loss to follow-up rate. Poor compliance was defined as taking <80% of pills.

### Outcomes

The primary outcome of the study was the *H. pylori* eradication rate 4 weeks after the end of treatment. Secondary outcomes included the rate of clinical symptom improvement (at the end of treatment and 4 weeks after the end of treatment), the rate of adverse events and the recurrence rate after eradication.

Due to the influence of COVID-19, some patients could not be reexamined at 4 weeks after the end of treatment, and the results of the breath test within 6 months after eradication treatment were finally determined.

### Calculation of sample size and statistical analysis

According to reports and previous studies (27, 28), furazolidone bismuth-containing quadruple therapy for 14 days demonstrated a high *H. pylori* eradication rate exceeding 90%. We assumed that the effective rate of each treatment group was the same as that of furazolidone bismuth-containing quadruple therapy for 14 days. In this study, each group receiving JWC was compared with the control group, with *α* = 0.05/3 = 0.017 and a power of 0.8. According to the clinically acceptable noninferiority level, the noninferiority margin value was −0.1, and the parallel control between the groups was designed 1:1:1:1. The sample size of the experimental group was 110 cases calculated by PASS 11.0 software. At a dropout rate of 10%, 122 patients per group were needed, resulting in a final requirement of 488 patients.

All statistical analyses were performed using IBM SPSS statistical software version 25.0 (IBM Inc., New York, USA) and SAS software, version 9.4 (SAS Institute Inc., Cary, NC, USA). Measurement data with a normal distribution are expressed herein as the mean ± standard deviation, and analysis of variance was used for comparisons between multiple groups of data. Measurement data with a nonnormal distribution were expressed asdescribed by the median (lower quartile, upper quartile). Count data and rank data were expressed as the number of cases and percentage. The chi-square test or Fisher’s exact test was used for the comparison of count data between groups, and the Kruskal–Wallis test was used for the comparison of multiple groups of rank data. The *H. pylori* eradication rate and the hypothesis of noninferiority were assessed based on ITT (including the participants who were enrolled in the study), MITT(including the participants who took at least one dose of medicine and underwent the endpoint measure), and PP(including the participants who were fully adherent with the protocol and excluding those with poor compliance) analyses. All tests were two-sided, and *p* < 0.05 was considered statistically significant.

## Results

**Figure 1:**
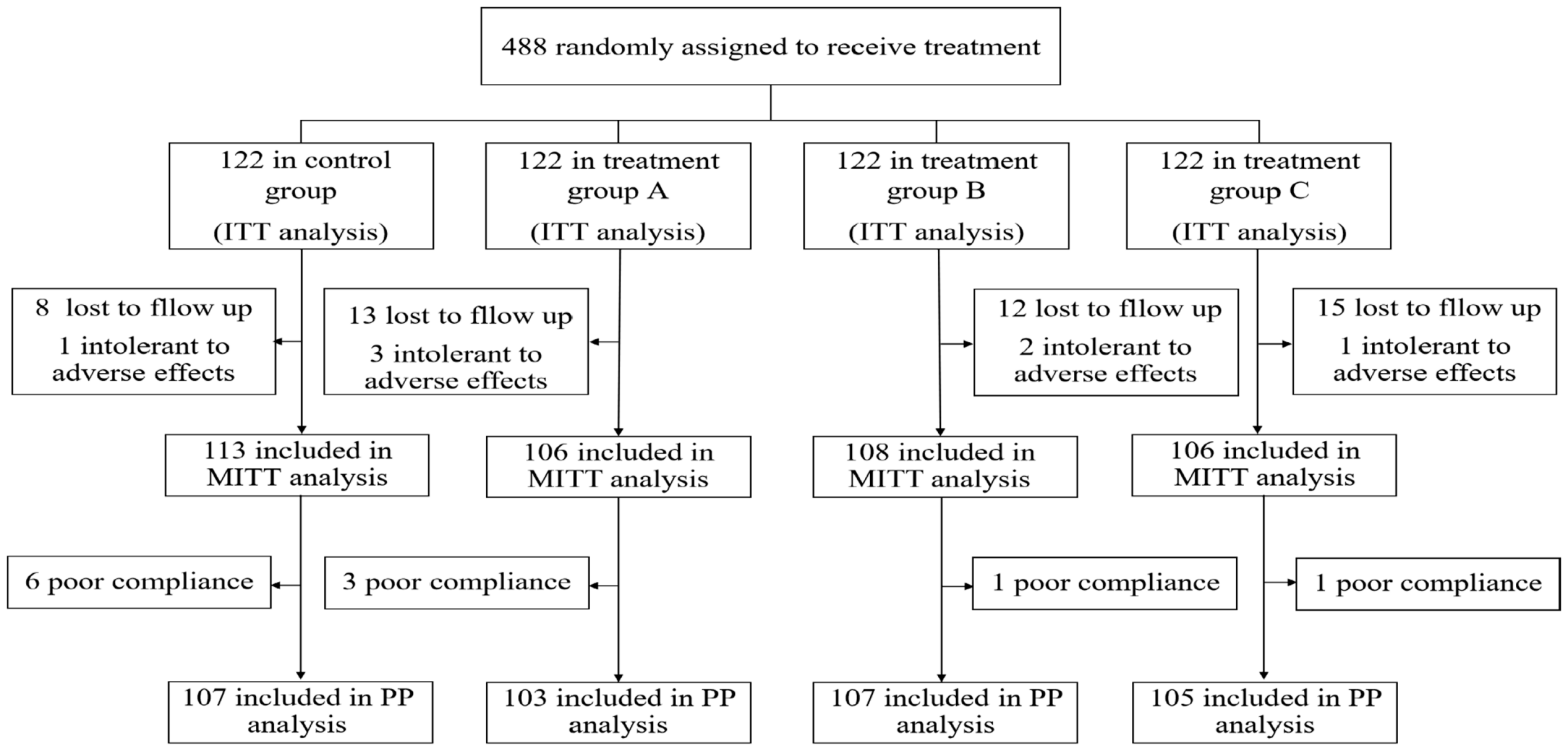
Flow diagram of the study.

As a result, a total of 488 patients were included in this study and randomized to receive quadruple and bismuth agent–containing JWC rescue treatment for *H. pylori* infection. All 488 participants were included in the ITT analysis. Participants who were lost to follow-up and those who had an intolerance for adverse events were excluded from the MITT analysis. In addition, excluding participants with poor adherence, the remaining patients were included in the PP analysis (Figure 1).

### Participant characteristics

There were no significant differences in the baseline characteristics, such as demographics, family history, number of eradications, and history of antibiotic treatment, among the four groups (all *p* > 0.05) (see Table 1).

**Table 1:** Baseline characteristics of the participants infected with *Helicobacter pylori* in whom eradication treatment had failed at least once.

Characteristics	Control group (*N* = 122)	Treatment group A (*N* = 122)	Treatment group B (*N* = 122)	Treatment group C (*N* = 122)	Statistics	*p* value
Age (years), mean ± SD	50.6 ± 11.8	50.5 ± 11.3	50.8 ± 10.9	50.5 ± 9.8	1.116^*^	0.341
male: female	52:70	50:72	44:78	52:70	1.462^†^	0.691
Symptom, *n* (%)					5.525^†^	0.137
Dyspepsia	101	108	95	97		
Others	21	14	27	25		
Family aggregation, *n* (%)	40	34	40	39	0.943^†^	0.815
Number of previous eradication attempts, *n* (%)					7.127^†^	0.309
1	87	87	79	75		
2	24	26	33	29		
≥3	11	9	10	18		
Previous antibiotic therapies (person-time)					5.271^†^	0.810
Amoxicillin + clarithromycin	79	74	84	66		
Amoxicillin + metronidazole	12	15	13	5		
Amoxicillin + levofloxacin	7	8	9	5		
Clarithromycin + levofloxacin	5	8	9	8		

Data are presented as mean ± standard deviation. SD: Standard deviation. ^*^ One-way Anova, ^†^*χ*^2^-value.

### Efficacy of *Helicobacter pylori* eradication therapy

A total of 433 patients underwent postmedication reexamination, with 414 patients reexamined at 4 weeks, 5 patients at 3 months, and the remaining patients at 6 months. ITT analysis showed that the eradication rate was 85.2% (104/122) in the control group and 73.8% (90/122), 78.7% (96/122) and 75.4% (92/122) in the treatment groups. In the MITT analysis, the eradication rates of the four groups were 92.0% (104/113), 84.9% (90/106), 88.9% (96/108), and 86.8% (92/106). PP analysis showed that the eradication rate was 92.5% (99/107) in the control group, 85.4% (88/103) in group A, 87.9% (94/107) in group B, and 86.7% (91/105) in group C. No statistically significant differences among the groups were found (*p* = 0.136, *p* = 0.398 and *p* = 0.405 for the ITT, MITT, and PP analyses, respectively), which suggests that the eradication rates of the three treatment groups are noninferior to the rate of the control group. The detailed results are shown in Table 2. Additionally, no statistically significant differences in eradication rates were observed between each of the three treatment groups and the control group (all *p* > 0.05), as detailed in Supplementary Tables 3, 4.

**Table 2:** *Helicobacter pylori* eradication rates in the four groups.

Items	Control group, % (n/N)	Treatment group A, % (n/N)	Treatment group B, % (n/N)	Treatment group C, % (n/N)	χ^2^-value	*P* value
ITT analysis	85.2 (104/122)	73.8 (90/122)	78.7 (96/122)	75.4 (92/122)	5.544	0.136
95% CI	85.2 (78.9–91.6)	73.8 (65.9–81.7)	78.7 (71.3–86.1)	75.4 (67.7–83.2)		
MITT analysis	92.0 (104/113)	84.9 (90/106)	88.9 (96/108)	86.8 (92/106)	2.958	0.398
95% CI	92.0 (87.0–97.1)	84.9 (78.0–91.8)	89.0 (83.0–95.0)	86.8 (80.2–93.3)		
PP analysis	92.5 (99/107)	85.4 (88/103)	87.9 (94/107)	86.7 (91/105)	2.916	0.405
95% CI	92.5 (87.5–97.6)	85.4 (78.5–92.4)	87.9 (81.6–94.1)	86.7 (80.1–93.3)		

CI, confidence interval.

### Non-inferiority analysis of *Helicobacter pylori* eradication rates

Non-inferiority analysis of *H. pylori* eradication rates, based on MITT analysis, were performed for each intervention group versus the control group. As summarized in Table 3, group B demonstrated non-inferiority with an eradication rate of 88.9% (*p* = 0.0415; 90% CI, −0.0965 to 0.0336). In contrast, group A exhibited an eradication rate of 84.9% (*p* = 0.2527; 90% CI, −0.1422 to 0.0004), and group C achieved an eradication rate of 86.8% (*p* = 0.1264; 90% CI, −0.1208 to 0.0160), neither of which met the prespecified non-inferiority margin.

**Table 3:** Non-inferiority analysis of *Helicobacter pylori* eradication rates.

Outcome	Control group	Treatment group A	Treatment group B	Treatment group C
Procedure success, % (n/N)	92.0 (104/113)	84.9 (90/106)	88.9 (96/108)	86.8 (92/106)
Risk difference	Reference	0.2527	0.0415^*^	0.1264
90% CI		(−0.1422–0.0004)	(−0.0965–0.0336)	(−0.1208–0.0160)

*P < 0.05; CI, confidence interval.

### Rates of adverse events, symptom improvement, and compliance

Among the 488 patients, 30 patients had adverse events, including 12 cases (9.84%, 12/122) in the control group, 7 cases (5.74%, 7/122) in group A, 8 cases (6.56%, 8/122) in group B, and 3 cases (2.46%, 3/122) in group C. There was no significant difference in the incidence of adverse events among the four groups (*p* > 0.05). The adverse reactions of the four groups all disappeared after drug withdrawal and other corresponding treatments. Details of the adverse reactions in 30 patients are shown in Table 4.

**Table 4:** Rates of adverse events in the four groups.

Items	Control group, *n* (%) (*N* = 122)	Treatment group A, *n* (%) (*N* = 122)	Treatment group B, *n* (%) (*N* = 122)	Treatment group C, *n* (%) (*N* = 122)	Overall numbers, *n* (%) (*N* = 488)	statistics	*P* value
Overall adverse events	12 (9.84)	7 (5.74)	8 (6.56)	3 (2.46)	30 (6.15)	5.864	0.112
Nausea	1 (0.81)	2 (1.63)	0	0	3 (0.61)	2.879	0.623
Fever	3 (2.46)	1 (0.81)	3 (2.46)	0	7 (1.43)	3.775	0.327
Skin rash	3 (2.45)	2 (1.63)	2 (1.63)	1 (0.81)	8 (1.64)	1.138	0.961
Abdominal Discomfort	2 (1.63)	2 (1.63)	1 (0.81)	1 (0.81)	6 (1.23)	0.914	1.000
Acroparaesthetia	1 (0.81)	1 (0.81)	1 (0.81)	0	3 (0.61)	1.476	1.000
Dizziness	2 (1.63)	1 (0.81)	2 (1.63)	1 (0.81)	6 (1.23)	0.914	1.000

As shown in Table 5, the four groups of patients with symptoms showed 69.8, 74.5, 80.9, and 86.3% improved effectiveness, respectively, at the end of treatment, and the differences among the four groups were statistically significant (*p* < 0.05). After 4 weeks of treatment, the effective improvement rates of symptoms in each group were 76.0, 85.7, 87.2, and 87.1%, respectively, which were not significantly different among the four groups (*p* > 0.05). There was also no significant difference in medication compliance (*p* > 0.05).

**Table 5:** Symptom improvement and compliance in the four groups.

Items	Control group, % (n/N)	Treatment group A, % (n/N)	Treatment group B, % (n/N)	Treatment group C, % (n/N)	*χ*^2^-value	*P* value
2-week symptom improvement	69.8 (74/106)	74.5 (73/98)	80.9 (76/94)	86.3 (88/102)	9.271	0.026^*^
4-week symptom improvement	76.0 (79/104)	85.7 (84/98)	87.2 (82/94)	87.1 (85/101)	6.637	0.084
Compliance rate	87.7 (107/122)	84.4 (103/122)	87.7 (107/122)	85.1 (105/122)	0.771	0.856

*P < 0.05.

We also compared improvements in major individual digestive symptoms (see Table 6). At the end of medication, the patients’ overall symptoms scores decreased. Among them, the curative effect of groups A, B and C was better than that of the control group in terms of bitter taste and dry mouth and abdominal pain, and the difference was statistically significant (*p* < 0.05). It can also be seen from Table 7 that four weeks after the end of medication, the symptoms of bitter taste and dry mouth in patients in groups A, B and C improved more significantly than those in the control group, and the symptoms of gastric noise of patients in groups B and C improved significantly, all of which were statistically significant (*p* < 0.05).

**Table 6:** Improvement of main single symptom score (2-week).

Items	Control group, % (n/N)	Treatment group A, % (n/N)	Treatment group B, % (n/N)	Treatment group C, % (n/N)	*c*^2^-value	*P* value
Abdominal distention	50.9% (27/53)	56.6% (30/53)	54.5% (30/55)	57.8% (37/64)	0.616	0.893
Abdominal pain	39.1% (18/46)	70.0% (28/40)	53.7% (22/41)	50.9% (27/53)	8.282	0.041^*^
Bitter and dry in mouth	49.3% (35/71)	50.0% (34/68)	50.8% (30/59)	70.1% (54/77)	9.133	0.028^*^
Belching	61.7% (37/60)	50.0% (34/68)	55.0% (33/60)	59.5% (44/74)	2.139	0.544
Clamor in the stomach	62.2% (28/45)	62.2% (23/37)	72.7% (24/33)	58.8% (30/51)	1.744	0.627

*P < 0.05.

**Table 7:** Improvement of main single symptom score (4-week).

Items	Control group, % (n/N)	Treatment group A, % (n/N)	Treatment group B, % (n/N)	Treatment group C, % (n/N)	*c*^2^-value	*P* value
Abdominal distention	61.5% (32/52)	67.3% (35/52)	57.7% (30/52)	58.5% (38/65)	1.292	0.731
Abdominal pain	50.0% (23/46)	69.0% (29/42)	62.5% (25/40)	69.2% (36/52)	4.849	0.183
Bitter and dry in mouth	51.9% (40/77)	61.2% (41/67)	67.8% (40/59)	77.0% (57/74)	10.930	0.012^*^
Belching	69.4% (43/62)	61.8% (42/68)	51.6 (32/62)	64.5% (49/76)	4.459	0.216
Clamor in the stomach	54.3% (25/46)	50.0% (23/46)	78.4% (29/37)	68.8% (33/48)	9.108	0.028^*^

*P < 0.05.

Some patients had no clinical symptoms before treatment, at the time of drug withdrawal, and 4 weeks after drug withdrawal, so they were not included in the calculation of the response rate. To ensure data integrity, we implemented online symptom inquiries for patients who were unable to attend follow-up appointments due to COVID-19.

### Recurrence rate after successful eradication

The follow-up for recurrence rates was conducted only with patients who tested negative on the breath test during reexamination of eradication 4 weeks after completing treatment. The *H. pylori* reinfection rate was higher in the control group than in the other three groups at 6 months after successful eradication, but the differences were not statistically significant (*p* > 0.05) (see Supplementary Table 1).

## Discussion

The Kyoto Global consensus on *H. pylori* gastritis recommends that all infected persons should be treated with eradication therapy if there are no counterbalancing factors. It is cost-effective to implement screening treatment to prevent gastric cancer in young people in areas associated with a high risk of gastric cancer (29). Although *H. pylori* resistance to antibiotics is becoming increasingly serious (30, 31), amoxicillin, clarithromycin, metronidazole, levofloxacin, tetracycline, furazolidone, etc., are still the antibiotics commonly used in *H. pylori* eradication therapy. Due to the limited research and development of new antibiotics, the screening of drugs with anti-*H. pylori* activity from traditional Chinese medicine and its components has become a key topic in the field of *H. pylori* treatment.

JWC is a Chinese patent medicine for the treatment of peptic ulcers and chronic gastritis that was developed by modern science and technology based on folk empirical prescription under the guidance of the theory of traditional Chinese medicine. JWC is composed of a volatile oil extracted from *Chenopodium* and *Rubiaceae* plants (32). Our previous studies have found that JWC can not only improve the level of gastric mucosa-related protective factors and inhibit or downregulate the expression of inflammatory factors but also significantly reduce gastric acid secretion and pepsin activity (33–37). JWC and its active component *Chenopodium ambrosioides* L. can reduce the adhesion of *H. pylori*, inhibit or destroy the biofilm formation of *H. pylori* (38), improve antibiotic sensitivity, kill sensitive and resistant strains of *H. pylori* (39–41), and have synergistic antibacterial effects when combined with antibiotics (42). JWC is widely used to treat *H. pylori* infection-related digestive system diseases. JWC combined with triple or quadruple therapy has a similar eradication rate of *H. pylori* as Western medicine alone, with more obvious improvement of clinical symptoms and fewer adverse reactions (43–46).

Considering that the treatment of traditional Chinese medicine requires a certain course of treatment to show curative effects, a group of schemes for prolonging the administration time of JWC was added to the treatment design. The total course of JWC treatment was 4 weeks. We also designed a treatment scheme of JWC combined with quadruple therapy for 10 days to more comprehensively observe the efficacy of JWC. ITT analysis, MITT analysis and PP analysis showed that although the eradication rate of JWC combined with triple or quadruple therapy was lower than that of bismuth quadruple therapy, there was no significant difference between the four groups. The group of regimen designed to prolong the administration time of JWC demonstrated non-inferiority in eradication rates. Therefore, for patients who cannot use bismuth due to objective factors, quadruple therapy containing JWC can be used to improve the eradication rate of *H. pylori*.

The main adverse reactions in this study were fever, rash, dizziness, nausea and vomiting, which were considered mainly related to antibiotics and bismuth. Most of the symptoms were mild and did not affect the treatment, and the adverse reactions of the remaining patients resolved upon discontinuation of medication and implementation of corresponding therapeutic measures. The common adverse reactions of furazolidone are nausea and dizziness, and peripheral nerve injury may occur in severe cases, which are related to the daily dose and total dose of furazolidone (47). Notably, only 3 cases of adverse events occurred in group C. One of the patients dropped out. The other two patients had mild symptoms and achieved successful eradication four weeks after the end of treatment. The regimen of group C was a 10-day course of low-dose furazolidone (200 mg/d), which not only achieved comparable *H. pylori* eradication rates to the 14-day bismuth quadruple therapy but also shortened the clinical treatment time, greatly reduced the exposure dose of antibiotics and bismuth, and reduced the incidence of antibiotic resistance and adverse reactions.

According to the Kyoto Global consensus on *H. pylori* gastritis, *H. pylori* gastritis is the cause of dyspepsia in some patients, and *H. pylori* eradication therapy should be the first choice for *H. pylori-*infected patients with dyspepsia symptoms. *H. pylori*-related dyspepsia should be considered in patients whose symptoms are relieved after eradication therapy (29). Abdominal pain, abdominal distension, belching, poor appetite and other symptoms are common dyspeptic symptoms in patients with chronic gastritis. In this study, after *H. pylori* eradication treatment, the related dyspeptic symptoms of patients in each treatment group were significantly improved, suggesting that *H. pylori* eradication therapy can improve the symptoms of dyspepsia in patients with chronic gastritis. Dyspepsia is associated with *H. pylori* infection. Traditional Chinese medicine holds that *H. pylori* is an external pathogen with dampness and heat. JWC can clear heat and dampness and regulate qi and dispel cold. Compared with the simple western medicine treatment, JWC can relieve the symptoms of abdominal pain, bitter taste and dry mouth and clamor in the stomach, and the difference was statistically significant. In terms of long-term efficacy, JWC showed the advantages of better symptom improvement while eradicating *H. pylori* infection.

At 6 months after successful eradication, there were reinfected patients in each group, and the control group had the highest recurrence rate, which was considered related to antibiotic resistance. Traditional Chinese medicine treatment emphasizes multitarget and holistic action. JWC is not simply antibacterial, and its mechanism of action may be related to regulating the gastrointestinal microecological environment and inhibiting inflammation (48).

The choice of *H. pylori* eradication regimen should be individualized. It is necessary to choose antibiotics reasonably according to the patient’s previous history of antibiotic use and the situation of *H. pylori* antibiotic resistance in the local population. JWC combined with amoxicillin-furazolidone triple/quadruple therapies demonstrated comparable eradication rates to the bismuth-containing quadruple therapy, with no statistically significant differences observed. Appropriate prolongation of the JWC administration duration demonstrated non-inferior efficacy in *H. pylori* eradication compared to bismuth-containing quadruple therapy, while effectively alleviating certain clinical symptoms and reducing the incidence of adverse events. It is suggested that for patients who are clinically contraindicated to receive bismuth, cannot use bismuth for other objective reasons or have obvious gastrointestinal symptoms, bismuth can be replaced by JWC, and by prolonging the administration time of JWC, the treatment effect on *H. pylori* infection can be further improved, and *H. pylori-*related symptoms can be improved more effectively. Bismuth-containing quadruple therapy combined with JWC for 10 days can be used for patients with severe clinical symptoms who cannot tolerate quadruple therapy, which can not only achieve an ideal *H. pylori* eradication rate but also reduce the incidence of adverse reactions.

JWC is suitable for the syndrome of mixed cold and heat, qi stagnation and blood stasis. This study did not carry out fine syndrome differentiation of the subjects. In the future, if we select more suitable patients for treatment on the basis of syndrome differentiation of traditional Chinese medicine, we may obtain a better curative effect. We did not perform antibiotic susceptibility testing, including for clarithromycin and amoxicillin. To minimize the risk of false-negative results, we strongly recommend completing a follow-up evaluation at the 6th month.

## Conclusion

The eradication efficacy of JWC combined with amoxicillin-furazolidone triple or quadruple therapy is comparable to that of bismuth-based quadruple therapy in the rescue therapy of *H. pylori*. Notably, the extended JWC administration regimen exhibits non-inferior therapeutic outcomes relative to the bismuth quadruple therapy group, while reducing the incidence of adverse events and demonstrating superior efficacy in alleviating specific clinical symptoms. Furthermore, the 10-day JWC combined with amoxicillin-furazolidone quadruple therapy effectively minimizes the exposure to antibiotics and bismuth, thereby decreasing the risk of adverse reactions. The therapeutic strategies hold considerable clinical significance, particularly for the eradication of antibiotic-resistant *H. pylori* in vulnerable populations, including elderly and pediatric patients, as well as those intolerant to quadruple therapy.

## Data Availability

The data analyzed in this study is subject to the following licenses/restrictions: the material and data that support the findings of this study are available from the corresponding author upon request. Requests to access these datasets should be directed to zhang.xuezhi@263.net.
