# Supplementary material for: Efficacy and safety of Jinghua Weikang capsule combined with amoxicillin-furazolidone triple/quadruple therapies in the rescue treatment of Helicobacter pylori infection
Source: Front Med (Lausanne). 2025 Mar 25;12:1531620. doi: 10.3389/fmed.2025.1531620 (PMC11975852; doi:10.3389/fmed.2025.1531620)
Supplement: Supplementary file 2 [file Data_Sheet_2.pdf]

Table S1

Table S1: *H. pylori* infection recurrence rates in the four groups (6-month)

Items	Control group, % (n/N)	Treatment group A, % (n/N)	Treatment group B, % (n/N)	Treatment group C, % (n/N)	χ^2-value	*P* value
6-month recurrence rate	11.8(9/76)	5.3(3/57)	3.1(2/65)	6.6(4/61)	4.586	0.198
95% CI	11.8(4.4-19.3)	5.3(-0.7-11.2)	3.1(-1.2-7.4)	6.6(0.2-12.9)		

CI: Confidence interval.

Table S2

The medication diary card for the control group is shown in the table below.

Days of medication	before breakfast Rabeprazole + bismuth	after breakfast Amoxicillin + furazolidone	before dinner Rabeprazole + bismuth	after dinner Amoxicillin + furazolidone
1				
2				
3				
4				
5				
6				
7				
8				
9				
10				
11				
12				
13				
14				

Note: Please take the medicine on time and mark "√" in the corresponding position in time. If there is any adverse reaction, please record.

Table S3

H. pylori eradication rates between each intervention group and the control group included in the PP analysis

Items	Control group	Treatment group A	Treatment group B	Treatment group C
Procedure success, % (n/N)	92.5(99/107)	85.4(88/103)	87.9(94/107)	86.7(91/105)
χ^2-value	Reference	2.7023	1.3200	1.9545
P value		0.1002	0.2506	0.1621

Table S4

H. pylori eradication rates between each intervention group and the control group included in the MITT analysis

Items	Control group	Treatment group A	Treatment group B	Treatment group C
Procedure success, % (n/N)	92.0(104/113)	84.9(90/106)	88.9(96/108)	86.8(92/106)
χ^2-value	Reference	2.7494	0.6358	1.5995
P value		0.0973	0.4252	0.2060

Types of study design

This study was a prospective multi-center randomized controlled clinical study. Randomization was designed by the Medical Statistics Department of Peking University First Hospital according to the central block method, and the allocation sequence table was prepared. At the same time, the allocation sequence list was handed to the personnel who were not involved in the screening,

intervention, follow-up and efficacy evaluation of the study subjects. All drugs were packaged according to the allocation plan. At the time of enrollment, medications were assigned according to the coding sequence, and the order of medication collection was not disrupted. The grouping results were confidential to the researchers and the subjects. Only after the subjects signed the informed consent, the carton was opened and the specific grouping of the patients could be known according to the intervention methods in the box, so that the allocation of the random plan could be concealed.
